# Two synchronous paraneoplastic endocrine syndromes in a 53-year-old male with broadly metastatic widely invasive Hürthle cell carcinoma

**DOI:** 10.1530/EDM-23-0118

**Published:** 2024-01-31

**Authors:** John J Orrego, Joseph A Chorny

**Affiliations:** 1 Department of Endocrinology and Metabolism, Kaiser Foundation Health Plan of Colorado, Denver, Colorado, USA; 2 Department of Pathology, Kaiser Foundation Health Plan of Colorado, Denver, Colorado, USA

**Keywords:** Adult, Male, White, United States, Thyroid, Thyroid, Endocrine-related cancer, Unique/unexpected symptoms or presentations of a disease, January, 2024

## Abstract

**Summary:**

Unlike medullary thyroid carcinomas, follicular cell-derived thyroid malignancies have rarely been associated with paraneoplastic endocrine syndromes. An ultrarare case of a middle-aged man with heavily treated broadly metastatic radioactive iodine-refractory widely invasive Hürthle cell carcinoma (HCC) of the thyroid with two synchronous paraneoplastic endocrine syndromes, T3 thyrotoxicosis and hypercalcemia of malignancy, is discussed here. The levothyroxine-induced T3 thyrotoxicosis was a gradual process that became more noticeable as the tumor burden, refractory to different modalities of therapy, expanded. The 1,25-dihydroxyvitamin-D-mediated hypercalcemia, on the other hand, developed in a manner of weeks, as it usually happens. It is important to emphasize that in patients with metastatic Hürthle cell and follicular carcinomas of the thyroid, on TSH suppressive therapy, the unexplained and progressive decline in FT4 and rise in FT3 levels, resulting in an elevated FT4/FT3 ratio, could be an indication of augmented type 1 (D1) and/or type 2 (D2) deiodinase expression in tumoral tissue, causing an increased conversion from the prohormone T4 into the active metabolite T3 via outer ring deiodination.

**Learning points:**

- Albeit extremely rare, some patients with thyroid cancer can present with more than one concomitant paraneoplastic syndrome.
- Although medullary thyroid carcinoma is the thyroid malignancy that is usually associated with paraneoplastic endocrine syndromes, follicular cell-derived thyroid cancers have been rarely described as being the culprit.
- In patients with metastatic Hürthle cell and follicular thyroid carcinomas, the unexplained and progressive decline in FT4 and rise in FT3 levels could be an indication of augmented type 1 (D1) and/or type 2 (D2) deiodinase expression in tumoral tissue, causing an increased conversion from T4 into T3 leading to T3 thyrotoxicosis.

## Background

Paraneoplastic syndromes (PNS) can affect up to 8% of patients with cancer (1). Lung, ovarian, and breast cancer, as well as lymphoproliferative disorders, are the leading causes of PNS, which are known to affect the endocrine, neurologic, dermatologic, rheumatologic, and hematologic organ systems. Paraneoplastic endocrine syndromes are usually associated with tumoral secretion of hormones or peptides (2). Most PNS associated with thyroid malignancies occur in patients with medullary thyroid carcinoma (MTC) (2). Follicular cell-derived thyroid carcinomas have rarely been linked to paraneoplastic endocrine syndromes. An ultrarare case of a 53-year-old man with heavily treated broadly metastatic radioactive iodine-refractory widely invasive Hürthle cell carcinoma (HCC) of the thyroid complicated by two synchronous paraneoplastic endocrine syndromes, T3 thyrotoxicosis and 1,25-dihydroxyvitamin-D-induced hypercalcemia, is described here.

## Case presentation

**Figure 1:**
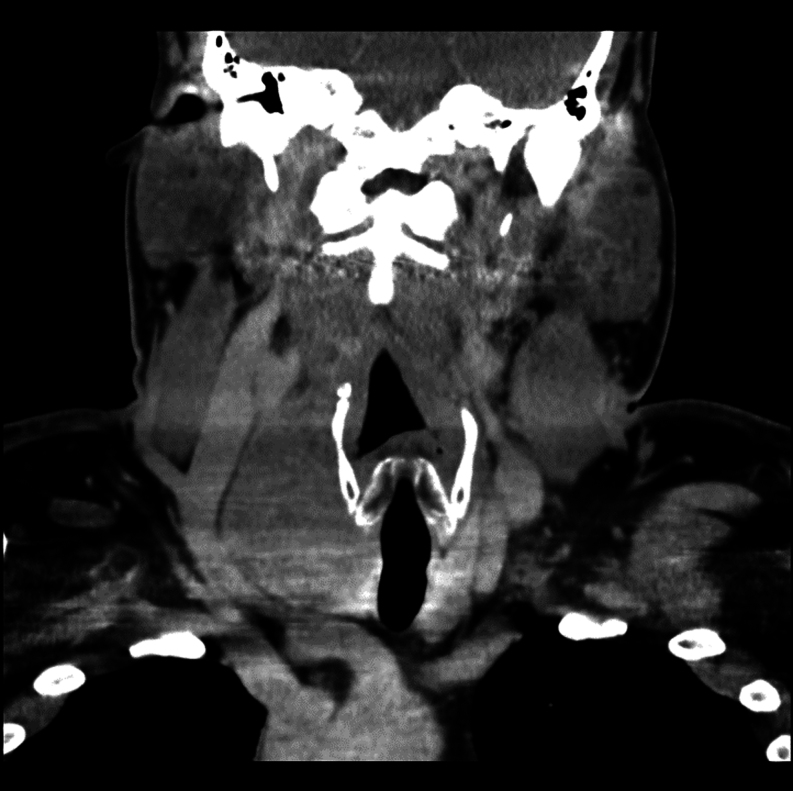
CT of the neck, performed after the administration of intravenous contrast material, revealed a 7.7 × 4.3 × 4.4 cm (cephalocaudal by transverse by AP dimensions) right thyroid mass with slight leftward displacement of the larynx and subglottic trachea.

A 46-year-old man with a right neck mass, which he had first noticed 3 months before presentation, was evaluated because of worsening snoring. He denied systemic or any other compressive symptoms. CT of the neck, performed after the administration of intravenous contrast material, demonstrated a 7.7 × 4.3 × 4.4 cm right thyroid mass, displacing the larynx and trachea to the left (Fig. 1). He was clinically and biochemically euthyroid. Ultrasound-guided fine needle aspiration (FNA) of this mass revealed a Hürthle cell neoplasm. ThyroSeq® Genomic Classifier testing demonstrated a TERT promoter mutation (C228T).

## Investigation, treatment, outcome, and follow-up

**Figure 2:**
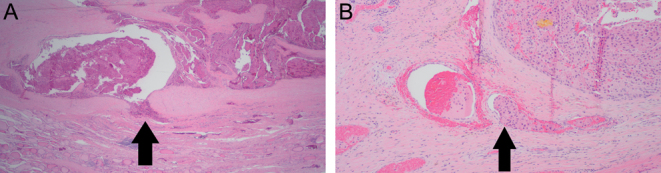
Pathology of the tumor. A. There is a Hürthle cell carcinoma with multiple foci of capsular perforation (arrow, H&E stain, 20×). B. lymphovascular invasion (arrow, H&E stain, 100×).

He underwent staged near-total thyroidectomy in 2016. Pathological examination of the resected specimens disclosed a 5.2 cm partially encapsulated HCC with extensive capsular and vascular invasion (Fig. 2). No lymph nodes were present in the excised surgical material. Levothyroxine 200 µg per day was prescribed, and 3 months later, the TSH was 0.031 µIU/mL, the FT4 was 1.8 ng/dL, the FT3 was 4.0 pg/mL, the thyroglobulin (Tg) was 40.2 ng/mL, and the Tg antibody (TgAb) was <0.9 IU/mL. The patient was treated with 141 mCi of radioactive iodine (^131^I) after levothyroxine withdrawal (the TSH was 39 µIU/mL), and posttreatment whole-body scan (WBS) imaging showed a small focus of increased activity overlying the left thyroid bed.

Fluorodeoxyglucose (FDG)-PET scan a year later showed interval development of few scattered sub-5 mm pulmonary nodules. Six months later, an FDG-PET scan demonstrated that the pulmonary nodules had increased in size and number and that some of them showed new mild FDG uptake. The largest one, in the right lower lung, measured 1.1 cm. The TSH level was 0.032 µIU/mL, the FT4 was 1.60 ng/dL, the FT3 was 3.53 pg/mL, the Tg was 843 ng/mL, and the TgAb was <0.9 IU/mL. ^131^I WBS with thyrogen showed no abnormal uptake. The patient was offered a clinical trial but opted against and chose complementary therapies.

**Figure 3:**
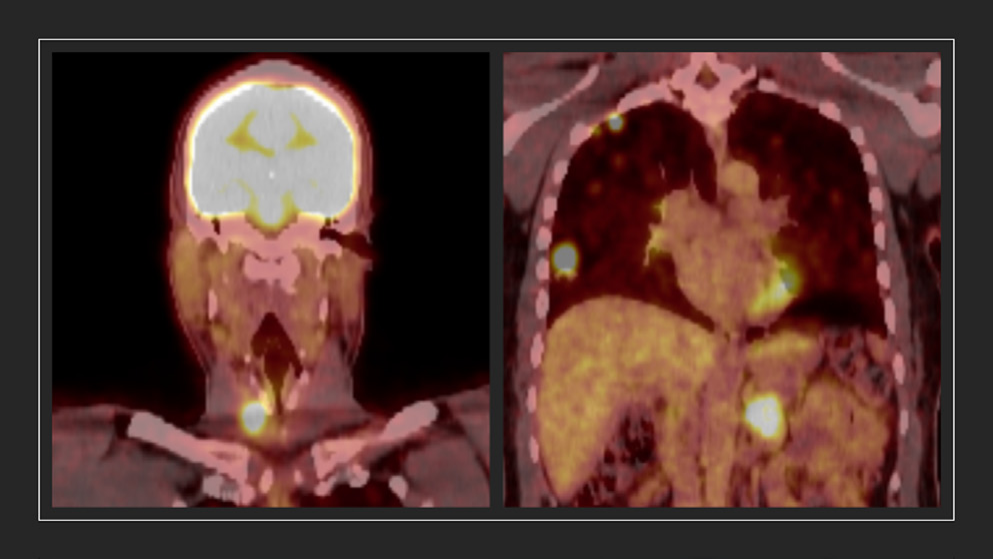
FDG-PET scan revealed a 3.0 × 1.7 cm (transverse by AP dimensions) right thyroid bed mass with an SUV_max_ of greater than 10, multiple metastatic pulmonary nodules, including a 2.0 × 1.8 cm in the medial right middle lobe, and a 3.2 × 3.4 cm left retroperitoneal mass with an SUV_max_ of 8.3.

FDG-PET scan 15 months later (Fig. 3) revealed a 3.0 × 1.7 cm right thyroid bed mass, enlargement of many of the innumerable metastatic pulmonary nodules, mediastinal adenopathy, a 3.2 × 3.4 cm left retroperitoneal mass, and new uptake within the medial left psoas muscle. Lenvatinib, up to 24 mg daily, was started.

Nine months later, an FDG-PET scan showed mixed changes, with an overall stable to a mildly increased burden of FDG-avid malignancy. FNA of the right thyroid bed mass disclosed metastatic HCC with the same gain-of-function mutation of the TERT promoter (C228T). Immunohistochemistry testing for mismatch repair (MMR) proteins revealed no loss of nuclear expression of DNA MMR proteins. Lenvatinib was discontinued.

**Figure 4:**
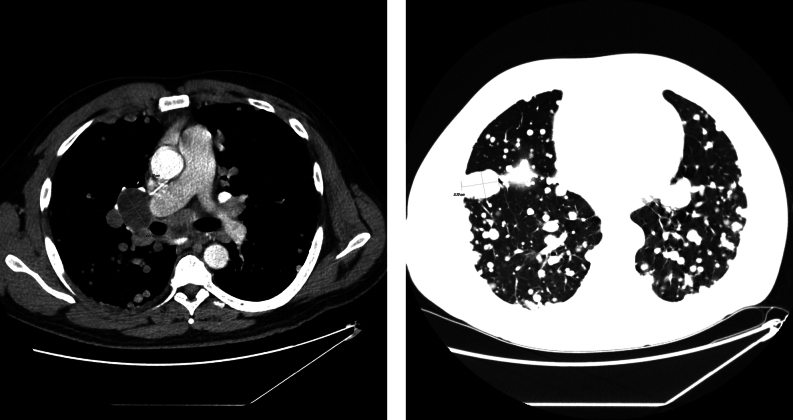
CT of the chest, performed after the administration of intravenous contrast material, revealed enlarging bilateral hilar lymphadenopathy, up to 2.6 × 3.5 cm on the right, and innumerable pulmonary nodules, up to 2.9 × 2.1 cm on the right mid lobe.

Six months later, the TSH was 0.01 µIU/mL, the FT4 was 1.04 ng/dL, the FT3 was 4.4 pg/mL, the Tg was >10 000 ng/dL, and the TgAb was <0.9 IU/mL. CT of the neck, chest, abdomen, and pelvis, performed after the administration of intravenous contrast material, revealed enlarging bulky right thyroid bed masses and mediastinal and bilateral hilar lymphadenopathy, innumerable pulmonary nodules, an enlarging 4.6 × 5.7 cm heterogeneous left retroperitoneal mass, and a 1.2 × 1.6 cm medial left psoas muscle metastasis (Fig. 4). Cabozantinib was started, which was discontinued after 4 months owing to worsening dyspnea associated with progressive pulmonary disease. Given an elevated FT3 of 6.6 pg/mL with undetectable TSH and an FT4 of 0.8 ng/dL, the levothyroxine dosing was decreased to 137 µg daily (Table 1).

**Table 1:** Pertinent levothyroxine (LT4) dosing and laboratory evaluations of the patient to depict the gradual and progressive change in the FT3/FT4 ratio from 2016 to 2023.

Date	LT4 (µg)	Tg (ng/mL)	TgAb (IU/mL)	TSH (µIU/mL)	FT4 (ng/dL)	FT3 (pg/mL)	FT3/FT4	rT3 (ng/dL)
July 15, 2016		31	<0.9	1.32				
November 4, 2019	175	>495	<0.9	0.035	1.4	4.15	2.96	
January 27, 2020	150			<0.008	1.1	3.17	2.88	
May 4, 2020	150	>495	<0.9	0.14	1.2	3.69	3.07	
September 3, 2020	150	>10 000	<0.9	0.01	1.04	4.4	4.23	
December 30, 2021	150	>495	<0.9	<0.008	0.8	6.6	8.25	
May 17, 2022	137			<0.008	0.5	7.49	14.98	
July 14, 2022	137			<0.005	0.4	4.69	11.72	
April 24, 2023	137	290.2	<0.9	0.009	0.2	5.83	29.15	<2.5
June 22, 2023	125			<0.005	0.22	3.38	15.36	
Reference range		1.3–31.8	0.0–4.0	0.32–5.5	0.7–1.8	2.3–4.2	3.2 ± 0.4	9.2–24.1

**Figure 5:**
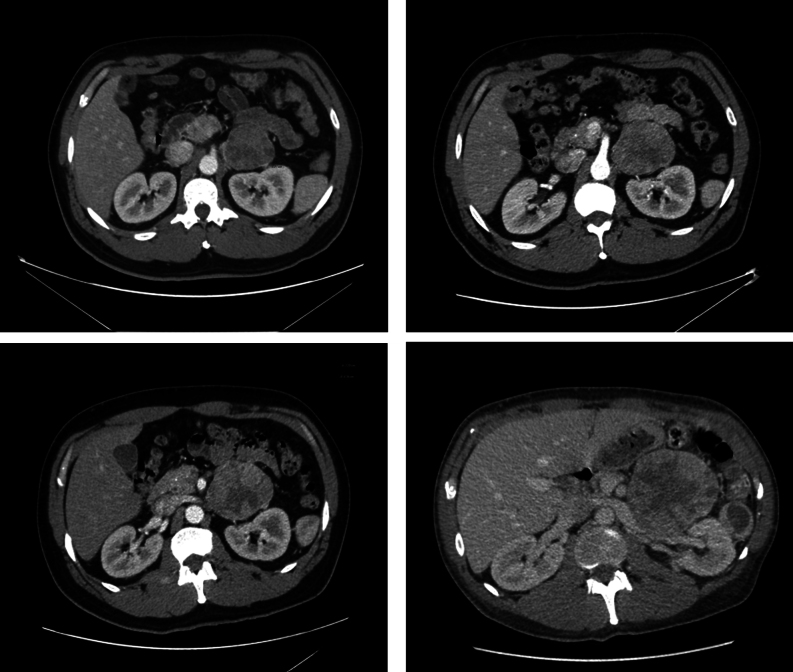
CT of the abdomen and pelvis, performed after the administration of intravenous material, demonstrated a progressively enlarging heterogeneous enhancing retroperitoneal mass thought to be arising from the tail of the pancreas, measuring up to 8.4 × 8.3 cm.

Pazopanib 600 mg daily was begun 6 months later, which was stopped after 7 months when a CT of the abdomen and pelvis, performed after the administration of intravenous material, demonstrated progressive disease, including an enlarging 8.4 × 8.3 cm heterogeneous enhancing retroperitoneal mass thought to be arising from the tail of the pancreas (Fig. 5) that was subsequently treated with palliative radiotherapy with 4000 cGy in ten fractions.

He developed severe hypercalcemia (14.2 mg/dL) 2 months later and was treated with zoledronic acid with transient normalization of his serum calcium. Subsequent work-up for hypercalcemia of malignancy revealed that the 1,25-dihydroxyvitamin D (calcitriol) was the causative factor (Table 2). Imaging studies revealed no bone metastases. An angiotensin-converting enzyme level was normal.

**Table 2:** Laboratory evaluations 10 weeks after the patient was admitted to the hospital with a hypercalcemic crisis.

Parameter	Value	Reference range
Calcium (mg/dL)	11.2	8.5–10.5
Albumin (g/dL)	3.7	3.3–4.8
PTH (pg/mL)	<6.0	14–84
Creatinine (mg/dL)	0.61	0.6–1.3
Phosphorus (mg/dL)	3.3	2.1–3.9
25(OH) vitamin D (ng/mL)	39	20–96
PTHrP (pg/mL)	11	14–27
Calcitriol (pg/mL)	137	18–72

Given the persistent elevation of the FT3 despite a very low FT4 level, the dose of levothyroxine was reduced from 137 to 125 µg per day with normalization of the FT3 but no change in the FT4 and TSH levels (Table 1).

At the time this case was written, he was under palliative care, had lost 72 lb over the preceding 9 months, and was oxygen dependent (6 L/min).

## Discussion

HCC of the thyroid is an uncommon subtype of thyroid malignancy, comprising ~5% of all well-differentiated thyroid cancers. Since 2017, HCC has been classified by the World Health Organization as a separate entity, instead of oncocytic variant of follicular thyroid carcinoma (FTC) (3). HCC is more common in elderly patients and men, can develop cervical lymph node metastasis, has higher-stage disease at diagnosis and worse prognosis, and is refractory to ^131^I (4).

A PubMed search without language restriction for publications containing the terms ‘paraneoplastic syndrome’ and ‘thyroid cancer’ up to July 2023 identified 463 references, of which 35 were follicular cell-derived thyroid neoplasms complicated by PNS. In this review, there were 28, 2, and 3 papillary, follicular, and poorly differentiated/anaplastic thyroid cancers, respectively, and 2 Hürthle cell neoplasms. Sixteen of the 35 patients (46%) with follicular cell-derived thyroid neoplasms had paraneoplastic neurologic syndromes, but only two of them (6%), a patient with PTC and SIADH and a patient with HC neoplasm and Cushing’s syndrome, had paraneoplastic endocrine syndromes.

Deiodinases are a family of three selenoenzymes that play a critical role in the regulation of intracellular and circulating concentrations of thyroid hormones. Type 1 (D1) and type 2 (D2) deiodinases convert the prohormone T4 into the active metabolite T3 via outer ring deiodination, while type 3 (D3) deiodinase inactivates T4 into reverse T3 (rT3) and T3 into T2 through inner ring deiodination (5). Normal thyroid follicular cells express D1 and D2 situated in the plasma membrane and endoplasmic reticulum, respectively. However, they do not express D3, an enzyme found exclusively in the plasma membrane. In Graves’ disease and toxic adenomas, the thyroid tissue’s expression of D1 and D2 is significantly increased compared with normal (5). Conversely, during thyroid tumorigenesis, the deiodinase expression profile changes according to the different needs of the malignant cells. While classic papillary thyroid cancer (PTC) can express D3 and downregulate D1 and D2, follicular thyroid carcinoma (FTC) and HCC can overexpress D1 and D2 (5).

Lang and Flesch described a patient with metastatic FTC with symptomatic T3 hyperthyroidism in the setting of normal-to-low T4 levels while on TSH suppressive therapy and they hypothesized that an increased 5′-deiodination from T4 to T3 was the culprit (6).

Kim *et al.* reported on three patients with FTC with increased serum T3–T4 ratio. Two of them had widely metastatic disease, and the other one had a massive primary tumor involving the right thyroid lobe and the mediastinum. The latter patient had both low FT4 and rT3 and normal FT3 and TSH levels. The FT4 and rT3 normalized when a transcervical, trans-sternal right thyroidectomy was performed to remove the tumor. D1 and D2 activities were tested in this 965 g specimen, and the V_max_ for D2 was eight-fold higher than in normal thyroid tissue (7).

Takano *et al.* described two patients with metastatic FTC with high circulating levels of FT3 but low to low-normal levels of FT4 while on TSH suppressive therapy. Quantitative measurement of D1 and D2 mRNAs, performed on samples from both the primary tumor and a distant metastasis of one of these patients, revealed increased expression of both deiodinases (8).

To investigate the prevalence of T3 thyrotoxicosis in athyreotic patients with widely metastatic thyroid cancer on TSH suppressive therapy, Miyauchi *et al.* identified 31 patients with PTC, 20 with FTC, and seven with MTC that were being treated at a single institution. Four patients with FTC (20%) but none in the other two groups exhibited T3 thyrotoxicosis and/or a FT3–FT4 ratio >3.5. D1 and D2 activities were measured on three frozen stored tumor tissues from two of these four patients, which demonstrated an 8- and 250-fold higher D1 and D2 activities, respectively, when compared with six normal control thyroid tissues obtained from the contralateral thyroid lobe without thyroid cancer (9).

Hypercalcemia of malignancy affects 20–30% of patients with cancer, with breast, lung, kidney, and squamous cell carcinomas, being the most common solid tumors causing this condition. Thyroid cancer has very rarely been associated with hypercalcemia of malignancy. Most cancer-associated hypercalcemia cases are due to tumoral secretion of parathyroid hormone-related peptide (PTHrP) and osteolytic metastases, but a minority are caused by tumoral secretion of 1,25-dihydroxyvitamin D.

A PubMed search, without language restriction, for publications containing the combination of terms ‘hypercalcemia’ and ‘thyroid cancer’, identified 646 references, of which seven were follicular cell-derived thyroid cancers complicated by hypercalcemia. Three patients with anaplastic thyroid carcinoma (ATC) developed PTHrP-mediated hypercalcemia (10, 11), and one of them also had marked leukocytosis that was mediated by granulocyte colony-stimulating factor (G-CSF) (12). One patient with PTC experienced hypercalcemia due to ectopic PTH secretion by the tumor (13), and another one, PTHrP-mediated hypercalcemia and CSF-mediated massive leukocytosis (14). One patient with functioning bone metastases from FTC developed local osteolytic hypercalcemia (15).

The seventh patient, a 69-year-old man with HCC metastatic to the liver, also experienced 1,25-dihydroxyvitamin-D-induced hypercalcemia and T3 thyrotoxicosis. He presented with a gradual lowering of his FT4 level over the preceding 9 years. He had undergone partial thyroidectomy 17 years before for what was thought to be a Hürthle cell adenoma of the thyroid. On no thyroid replacement therapy, the FT4 and FT3 levels had become undetectable and slightly elevated, respectively, while the TSH level remained normal. The 20 cm liver mass was deemed to be irresectable. He underwent a complete thyroidectomy followed by ^131^I therapy. The posttreatment WBS was negative. The patient developed intractable severe hypercalcemia complicated by renal insufficiency, which finally caused his demise. The PTH was low, PTHrP was undetectable, and the 1,25-dihydroxyvitamin D was elevated at 258 pmol/L (normal: 55–138 pmol/L). D1 and D2 activities in primary or metastatic tumoral tissue were not assessed (16).

A patient with broadly metastatic, widely invasive HCC of the thyroid with two synchronous paraneoplastic endocrine syndromes, T3 thyrotoxicosis and hypercalcemia of malignancy, is presented here. In retrospect, the first sign of T3 thyrotoxicosis can be traced back to September 2020, which was 4.5 years after the diagnosis of widely invasive HCC had been made. Although D1 and D2 activities were not assessed in the resected thyroid and metastatic tissue, it is hypothesized that the elevated FT3 and the gradual and progressive lowering of the FT4 levels in the setting of progressive disease and increased tumor burden are consistent with increased D1 and/or D2 activity. The undetectable rT3 and the normalization of the FT3 with lowering of the levothyroxine dosing are also in favor of this hypothesis.

The hypercalcemia of malignancy, on the other hand, was detected close to 7 years after total thyroidectomy and probably developed within a matter of weeks. This type of hypercalcemia was likely due to upregulation of the expression of *Cyp27B1* in tumor cells, which encodes 1-α-hydroxylase, the enzyme responsible for converting 25-hydroxyvitamin D to the active hormone 1,25-dihydroxyvitamin D. Excess 1,25-dihydroxyvitamin D increases intestinal calcium absorption as well as bone resorption, leading to hypercalcemia.

It is important to emphasize that in the differential diagnosis of ‘weird’ thyroid function tests, aside from drugs, supplements, poor compliance, nonthyroidal illness, and assay interference, changes in the expression patterns of D1 and/or D2 activity in normal or diseased thyroid tissue should be included.

## Declaration of interest

The authors declare that there is no conflict of interest that could be perceived as prejudicing the impartiality of the case study reported.

## Funding

This work did not receive any specific grant from any funding agency in the public, commercial, or not-for-profit sector.

## Patient consent

Written informed consent was obtained from the patient for publication of this case report.

## Author contribution statement

JJO (endocrinologist) interviewed and examined the patient and ordered all pertinent tests, and JAC (pathologist) diagnosed the endocrine lesions. Both contributed to writing the manuscript.
